# Effect of Ethyl-Cyanoacrylate and Platelet-Rich Fibrin on Fresh Sockets of Rabbits Subjected to Anticoagulant Therapy

**DOI:** 10.3390/jcm13216389

**Published:** 2024-10-25

**Authors:** Eduardo Rosas, Fernando José Dias, Dimitrius Pitol, Sergio Olate, João Paulo Mardegan Issa, Eduardo Borie

**Affiliations:** 1 Master Program in Dental Sciences, Dental School, Universidad de La Frontera, Temuco 4811230, Chile; e.rosas02@ufromail.cl; 2 Oral Biology Research Centre (CIBO-UFRO), Adults Integral Dentistry Department, Dental School, Universidad de La Frontera, Temuco 4811230, Chile; fernando.dias@ufrontera.cl; 3 Department of Basic and Oral Biology, School of Dentistry of Ribeirão Preto, University of São Paulo, Ribeirão Preto 14040-900, SP, Brazil; dimitrius@forp.usp.br (D.P.); jpmissa@forp.usp.br (J.P.M.I.); 4 CIMA Research Centre, Adults Integral Dentistry Department, Dental School, Universidad de La Frontera, Temuco 4811230, Chile; sergio.olate@ufrontera.cl; 5 Center of Excellence in Morphological and Surgical Studies (CEMyQ), Universidad de La Frontera, Temuco 4811230, Chile; 6 CICO Research Centre, Adults Integral Dentistry Department, Dental School, Universidad de La Frontera, Temuco 4811230, Chile

**Keywords:** cyanoacrylate, platelet-rich fibrin, sockets, anticoagulant, rabbits

## Abstract

**Objectives**: There are no studies related to the use of PRF associated with cyanoacrylates in fresh post-extraction sockets. Thus, the aim of this study was to assess the effect of ethyl-cyanoacrylate combined with PRF in fresh sockets of rabbits subjected to anticoagulant therapy. **Methods**: Twelve adults rabbits were selected and premedicated with heparin 1 week before surgery to induce and simulate anticoagulant therapy. Upper and lower first premolars on the right side were extracted and then were divided into four groups of three animals each, with the groups distributed according to the type of intervention in the sockets (*n* = 6): (1) clot and suture (control); (2) PRF and suture; (3) clot and ethyl-cyanoacrylate; (4) PRF and ethyl-cyanoacrylate. At 12 weeks, the animals were sacrificed and the sockets were analyzed histologically and quantitatively. Total bone area, inflammation infiltrate, and adhesive remnants were assessed. **Results**: No remnants of adhesive were found in the samples. Groups 1 and 2 showed the highest bone area (G1 = 37.87% ± 17.86; G2 = 30.31 ± 9.36) with significant differences to those treated with ethyl-cyanoacrylate adhesive (G3 = 26.6% ± 11.82; G4 = 24.29% ± 6.25). **Conclusions**: The groups that used ethyl-cyanoacrylate as a closure method in sockets exhibited less bone area than the groups that used sutures. Both groups that used PRF as therapy did not show a significant improvement in bone healing at 12 weeks compared with the clot groups.

## 1. Introduction

Ethyl-cyanoacrylate, one of the shortest-chain cyanoacrylate adhesives, was among the first adhesives tested for clinical use [1] and has shown good biocompatibility in human osteoblast cell cultures [2]. Its excellent adhesive strength, stability, rapid polymerization in a moist environment, and adequate working time promote its use in both medicine and dentistry [3]. The literature reports several advantages of cyanoacrylate adhesives in oral surgery procedures [3], including closure methods for mandibular third molar extraction [4], free gingival graft surgery [5], bone graft fixation [6], and apical surgery [7], among others.

Cyanoacrylate adhesives are a good option for patients who have undergone anticoagulant therapy. Muzumdar and Feng [8] reported their use as a hemostatic therapy on the skin after dermatologic surgery in elderly patients. Borie et al. [3] reported the application of ethyl cyanoacrylate adhesive during a dental implant placement procedure to achieve immediate hemostasis in a patient undergoing anticoagulant therapy. Additionally, the use of cyanoacrylate adhesives as a hemostatic agent has been reported in patients undergoing anticoagulant therapy during multiple exodontia [9].

On the other hand, platelet-rich fibrin (PRF) is a beneficial tool for obtaining concentrated growth factors from the patient themselves, prepared through centrifugation without the need to add any chemicals [10]. PRF significantly improves bone and soft tissue regeneration. Over the last decade, its use has increased consistently, along with the number of published papers related to various clinical applications of PRF, with a high level of scientific evidence [11]. However, the use of PRF in fresh sockets to prevent bone loss and promote bone healing remains uncertain. Some studies [12,13] have found that PRF results in reduced bone volume loss compared to spontaneous healing in alveolar ridge preservation. Nevertheless, other studies [14,15] conclude that PRF does not reduce the rate of ridge resorption in either the vertical or horizontal dimensions of extraction sockets, nor does it induce greater new bone formation.

The association between PRF and cyanoacrylates has been studied in soft tissue management, such as PRF stabilization in free epithelialized mucosal grafts as well as the palatal donor site, observing promising results [16,17,18,19,20]. However, there are no studies related to the use of PRF associated with cyanoacrylates in fresh post-extraction sockets. Thus, the aim of this study was to assess the effect of ethyl-cyanoacrylate combined with PRF in fresh sockets of rabbits subjected to anticoagulant therapy. The hypothesis was that the combination of ethyl cyanoacrylate and PRF in fresh sockets of rabbits undergoing anticoagulant therapy would improve bone regeneration.

## 2. Materials and Methods

The research was approved by the Universidad de La Frontera Ethics Committee (Nº 126/21).

Twelve 7-month-old, healthy male rabbits (*Oryctolagus cuniculus*) with an average weight of 2.5 kg (±0.2 kg) were selected [21]. The care and handling of the animals were carried out following the ethical standards of the “Guide for the Care and Use of Laboratory Animals” [22] in the animal surgery room at the Bioterium of the Center of Excellence in Morphological and Surgical Studies (CEMYQ) of the University of the Frontera, Temuco, Chile. All rabbits were kept in the animal room with free access to water and food, at a temperature of 23–25 °C, humidity of 50–60%, and a 12 h light/dark cycle [23]. The animal room was clean, dry, and ventilated.

The rabbits were premedicated before surgery with an injection of heparin 30 IU/kg into the marginal auricular vein [24] once daily for 1 week to induce and simulate anticoagulant therapy. One hour before surgery, amoxicillin (50 mg/kg) was administered as a prophylactic antibiotic via intramuscular route. For sedation of the animals, a dose of Ketamine (40 mg/kg i.m.) supplemented with Xylazine (5 mg/kg i.m.) was used. In addition, as a local anesthetic, 0.4 mL of 2% lidocaine with a dose of 1:100,000 epinephrine (Octocaine-100, Cambridge, ON, Canada) was applied using an infiltrative technique of the upper and lower first premolars on the right side. Tooth extraction was only performed by one dentist with more than five years of experience and previously calibrated to obtain a clean extraction without root fracture, according to Hamad et al.’s [23] technique, where the first premolar is extracted, and once luxated, the alveolus is compressed to destroy the germ and thus prevent the growth of another tooth. To control the risk of infection in the animals, enrofloxacin (5 mg/kg i.m.) was indicated, while meloxicam (0.4 mg/kg i.m.) [25] was used for pain management.

Once the tooth extraction procedure was completed, the rabbits were divided into four groups of three animals each, with the groups distributed according to the type of intervention in the sockets (*n* = 6), which were as follows: (1) clot and suture (control); (2) PRF and suture; (3) clot and ethyl-cyanoacrylate; (4) PRF and ethyl-cyanoacrylate.

In both groups of sutures, absorbable sutures were used (Vicryl^®^, Novartis Animal Health US, Inc., Greensboro, NC, USA). In the groups that used ethyl-cyanoacrylate adhesive (Superbonder Loctite^®^, Düsseldorf, Germany), it was applied directly in an attempt to close the edges and occlude the alveolar bed. In the groups that used platelet-rich fibrin (PRF), it was obtained from a 1.8 mL sample of auricular venous blood in red tubes without biochemical additives. Immediately after obtaining the blood, this sample was centrifuged for 10 min at 3000 rpm in a clinical centrifuge (EBA 200, Kirchlengern, Germany) according to the protocol previously described [26,27].

Animals were euthanized with an overdose of sodium pentobarbital i.m. (60 mg/kg) at 12 weeks [21]. After euthanasia, the experimental sockets were removed with a security margin using an electric saw.

Sample processing and histological analysis were performed according to the protocol described by Ricardo et al. [28]. The alveolar bone samples were immersed in formaldehyde 4% for 24 h, followed by decalcification with EDTA + TRIS 0.5 M, which was changed every 2 days. The samples were dehydrated in a crescent alcohol series, diaphanized in xylol, and embedded in paraffin [28]. There were performed sample sections with 6 μm thickness stained with Masson trichrome for morphological evaluation. The qualitative analysis was performed with a light microscope (DM 4000B, Wetzlar, Germany) equipped with a digital camera (DFC310FX, Wetzlar, Germany).

Morphometric quantification was performed with ImageJ software (1.53e, NIH, Bethesda, MD, USA) in images with X10 magnification, applying a grid-like test system, with a total of 130 points applied to the photomicrographs obtained [28,29]. Total bone area, inflammation infiltrate, and adhesive remnants were analyzed.

The statistical analysis was performed using the “SigmaPlot 15.0” software. The normality of the data was confirmed by the Shapiro–Wilk test. Therefore, to compare the quantitative results, the one-way ANOVA test was applied with the Holm–Sidak post hoc test, allowing the analysis of multiple groups (data, mean ± SD, standard error of the mean).

## 3. Results

Regarding groups 3 and 4, where ethyl cyanoacrylate adhesive was applied, hemostasis was immediate. In contrast, in groups 3 and 4, which received resorbable sutures, achieving hemostasis took up to 3 min.

**Figure 1:**
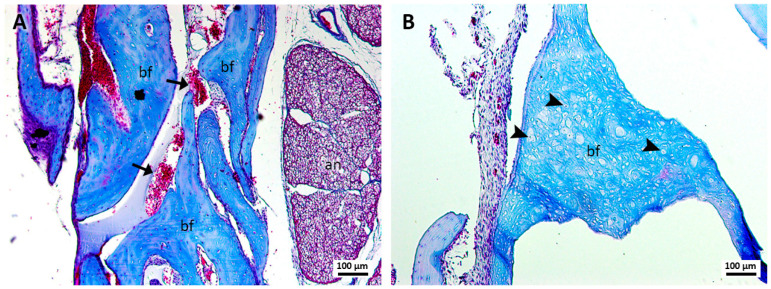
(**A**,**B**). Histological analysis of the sockets at 12 weeks. G1—suture and clot—robust bone formation (bf), presence of blood vessels (arrows), osteoblasts (arrowheads), and the alveolar nerve (an) were observed. Mag.: ×10.

Group 1, treated with sutures and blood clots (Figure 1A,B), revealed robust bone formation with apparently more regular structures compared to groups treated with cyanoacrylate (group 3) and cyanoacrylate + PRF (group 4). The newly formed bone in this group revealed a large number of blood vessels in the region of bone formation. In addition, areas of bone formation were observed in which a large number of osteoblasts were observed. In this group, the formation of loose connective tissue with areas suggestive of adipose formation was not so evident.

**Figure 2:**
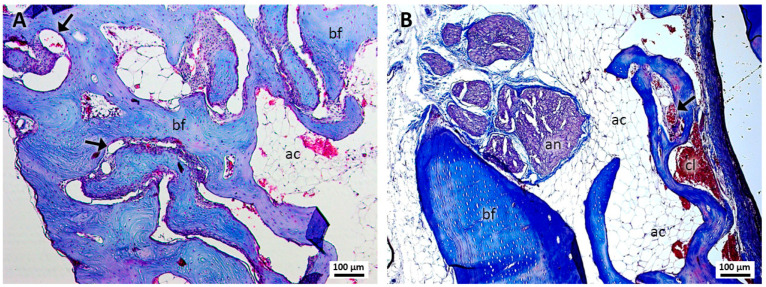
(**A**,**B**). Histological analysis of the sockets at 12 weeks. G2—PRF and suture—robust bone formation (bf), presence of blood capillaries (arrows), lax tissue similar to adipose cells (ac), the alveolar nerve (an), and structures suggestive of a blood clot (cl) were noted. Mag.: ×10.

In group 2, treated with PRF and sutures (Figure 2A,B), robust bone formation was revealed with the presence of massive osteocytes and some blood capillaries. The presence of lax tissue similar to adipose cells was evident, as well as erythrocytes and structures suggestive of clots in the region of the dental alveolus.

**Figure 3:**
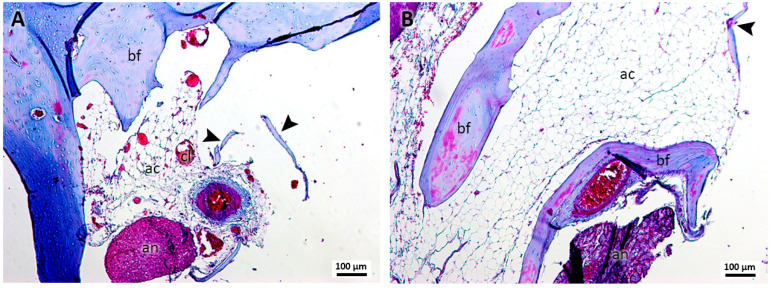
(**A**,**B**). Histological analysis of the sockets at 12 weeks. G3—ethyl-cyanoacrylate and clot—It is possible to observe bone formation (bf), bone islets (arrowhead), structures similar to adipose cells (ac), structures similar to blood clots (cl), and the alveolar nerve (an). Mag.: ×10.

In group 3, treated with cyanoacrylate and clots (Figure 3A,B), no remnants of this material were observed in the samples, identifying little bone formation with osteocytes and the presence of few and small bone islets. The presence of loose connective tissue with characteristics similar to those of adipocytes was evident, in addition to the presence of formations suggestive of blood clots.

**Figure 4:**
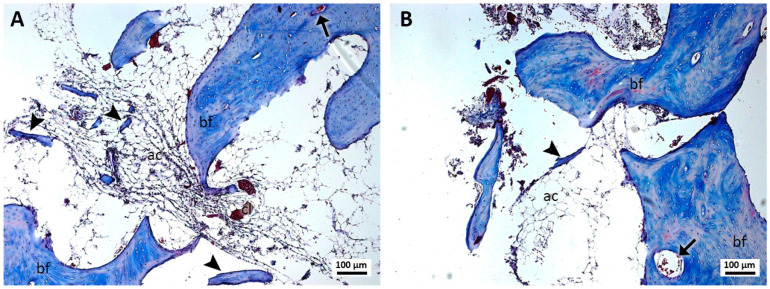
(**A**,**B**). Histological analysis of the sockets at 12 weeks. G4—ethyl-cyanoacrylate and PRF—bone formation (bf), bone islets (arrowhead), blood capillaries (arrow), structures similar to fat cells (ac), and structures similar to blood clots (cl) can be observed. Mag.: ×10.

In the samples of group 4, treated with cyanoacrylate and PRF (Figure 4A,B), the presence of PRF and cyanoacrylate was not identified. Bone formation was observed with the presence of osteocytes and a few small bone islets. The bone tissue showed capillaries and few cells suggestive of osteoblasts trapped by the structure. In the region of loose connective tissue, the presence of structures suggestive of clots and formations of adipose tissue was observed.

Considering that neither areas with inflammatory infiltrate nor remains of ethyl cyanoacrylate were observed in the samples from the four groups analyzed, quantification and comparisons only of the area of bone tissue formed were performed.

**Figure 5:**
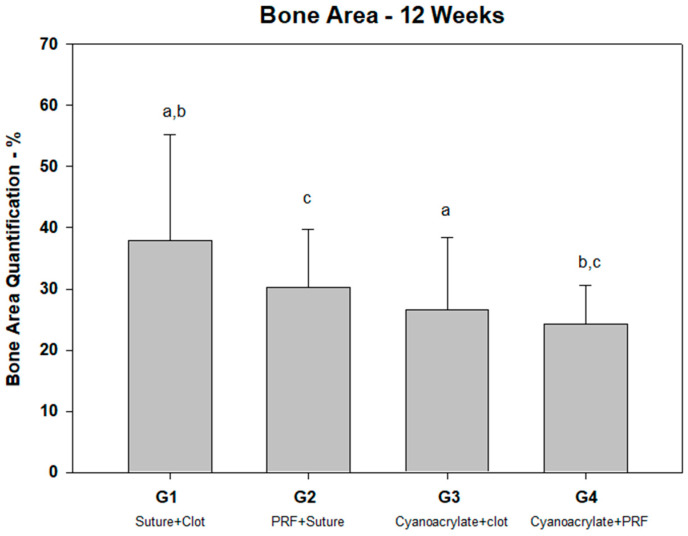
Percentage of bone area quantification. Note the differences between groups: a (*p* = 0.018); b (*p* = 0.01); c (*p* =0.037).

Regarding the bone tissue area (Table 1), the control group (clot and suture) showed a higher bone area (37.87% ± 17.86) together with the PRF and suture group (30.31 ± 9.36), without observing significant differences between both groups (Figure 5). On the other hand, the groups treated with ethyl-cyanoacrylate adhesive showed the lowest total bone area, without significant differences between them (G3 = 26.6% ± 11.82 vs. G4 = 24.29% ± 6.25); however, the PRF and ethyl-cyanoacrylate group showed significant differences with both the groups treated with resorbable sutures.

## 4. Discussion

The hypothesis was rejected due to the fact that ethyl-cyanoacrylate combined with PRF in fresh sockets of rabbits subjected to anticoagulant therapy did not improve bone regeneration.

Some studies support the use of ethyl-cyanoacrylates in bone healing, concluding that they are biocompatible with osteoblastic cells [2] and do not interfere with the process of bone healing and graft integration [6,30,31]. However, the results observed in our study demonstrated less bone formation in the groups where ethyl-cyanoacrylate was used. In this context, several studies support our findings. A study of ethyl-cyanoacrylate as a bone graft fixation method [32] demonstrated no graft integration during 120 days of follow-up in rabbit calvaria. A similar pilot study [33] involving autologous bone grafts in rabbits demonstrated the compatibility of ethyl-cyanoacrylate but not its effectiveness since there was no total integration of the graft. Other studies on rats showed similar results with cyanoacrylate adhesives, observing no graft fixation, no signs of bone apposition, and the absence of newly formed bone tissue [34,35,36].

In contrast from these last studies, the presence of inflammatory infiltrate and cyanoacrylate remnants were not identified. Some studies [1,30,32,33,34] found remnants of cyanoacrylates in their histological description, with some of them even preventing revascularization [34,36]. It is reported that the degradation of the cyanoacrylate adhesive can take up to 48 weeks [37]. Tzagiollari et al. [38] report that one of the major disadvantages of synthetic adhesives such as cyanoacrylates is related to their biocompatibility and, mainly, their biodegradability. The difference observed in our study may be explained by the nature of the socket, which is an open cavity in constant contact with the tongue, tissues, and food, potentially leading to the dislodgment of the adhesive during the follow-up period.

Also, in both groups using ethyl-cyanoacrylates adhesives as a closure method, the presence of structures suggestive of clots and adipose tissue formation were observed, similar to the reports of Saska et al. [32]. The findings suggest that adipose tissue could be related to mesenchymal precursors and could differentiate into osteoblasts or adipocytes depending on the transcription factors [39]. In this sense, the presence of ethyl-cyanoacrylate could have favored adipogenesis and consequently inhibited osteoblastogenesis.

In the groups in which sutures were used as a closure method, robust areas of bone formation were observed in which a large number of osteoblasts were identified. This result is completely opposed to our initial expectations, as we hypothesized that the ethyl-cyanoacrylate adhesive would allow the stabilization of the blood clot or the PRF, allowing for alveolar bone neoformation due to the presence of growth factors; however, both groups in which ethyl-cyanoacrylate adhesive was used showed significantly less bone neoformation than the clot and suture group, which means it can be inferred that this adhesive negatively influenced the alveolar repair process.

There were no significant differences in the total bone area between the sockets treated with PRF and those treated with clots at 12 weeks. Platelet-rich fibrin seems to be important in the bone healing process only during the early period [40]. Matsumura et al. [41] identified only significant differences in bone healing in dog sockets at 2 weeks. Recent studies [42,43] on rabbit sockets observed differences in terms of the rate and amount of new bone formation compared with the control at 2 weeks, without observing differences at 4 and 8 weeks [43]. However, Damayanti and Rachmawati [44] compared the inhibition of MMP-13 in rabbits 3–7 and 14 days after tooth extraction, reporting no differences between the group treated only with PRF and blood clots.

One of the most clinically notable aspects is the immediate hemostasis achieved in both ethyl-cyanoacrylate groups, which concurs with the findings of Xavier et al. [30], who noted a fast polymerization of the adhesive taking up to 30 s. However, this great feature is not comparable with the poor histological findings in both groups that used ethyl-cyanoacrylate.

Among the limitations of our research are the single evaluation time (12 weeks), the low sample size, and the difficulty of controlling the sockets concerning food and friction; however, the findings are interesting regarding the possible inhibition of bone regeneration in the groups in which ethyl-cyanoacrylate was used.

Although this is an animal study, histological findings suggest that ethyl-cyanoacrylate negatively affects bone regeneration in rabbit sockets. Therefore, its use in patients is not recommended until future studies prove otherwise. However, further studies involving other types of tissue adhesives are necessary to determine whether ethyl-cyanoacrylate specifically interferes with bone regeneration in sockets, or if the entire family of cyanoacrylates, including butyl and octyl-cyanoacrylate, affects it too.

## 5. Conclusions

The groups that used ethyl-cyanoacrylate as a closure method in sockets exhibited less bone area than the groups that used sutures. Both groups that used PRF as therapy did not show a significant improvement in bone healing at 12 weeks compared with the clot groups.

## Figures and Tables

**Table 1:** Mean, standard deviation (SD), and standard error of the mean (SEM) of total bone area measured in the different groups.

Group	Mean	SD	SEM
G1—Suture and clot	37.87%	17.36	4.21
G2—PRF and suture	30.31%	9.36	1.80
G3—Ethyl-cyanoacrylate and clot	26.6%	11.82	2.41
G4—Ethyl-cyanoacrylate and PRF	24.29%	6.25	1.67

## Data Availability

The original contributions presented in the study are included in the article, further inquiries can be directed to the corresponding author.
